# Life-Threatening Hyponatremia, Hyperkalemia, and Shock in Infancy: Not Always Congenital Adrenal Hyperplasia

**DOI:** 10.7759/cureus.89285

**Published:** 2025-08-03

**Authors:** Wai Hung Chung, Manson Chon In Kuok, Kwai Yu Winnie Chan

**Affiliations:** 1 Department of Paediatrics, Queen Elizabeth Hospital, Hong Kong, HKG

**Keywords:** congenital anomalies of the kidney and urinary tract (cakut), hyperkalemia, hyponatremia, pseudohypoaldosteronism, urinary tract infection

## Abstract

Hyponatremia and hyperkalemia, though uncommon in infancy, are potentially life-threatening electrolyte disturbances. We discuss a case of secondary pseudohypoaldosteronism (PHA) in a six-month-old male presenting with recurrent vomiting, severe hyponatremia (119 mmol/L), hyperkalemia (6.6 mmol/L), and metabolic acidosis (bicarbonate: 9 mmol/L). The patient’s condition rapidly deteriorated with hypotension and respiratory distress requiring intensive care and mechanical ventilation. Investigations identified an Escherichia coli urinary tract infection (UTI), and further laboratory tests revealed markedly elevated serum renin and aldosterone levels, consistent with secondary PHA associated with UTI in infancy. This report explores the incidence, proposed pathophysiology, and management strategies for secondary PHA. Additionally, we provide information to aid in differentiating among the potential causes of similar biochemical abnormalities, including congenital adrenal hyperplasia (CAH), in pediatric patients.

## Introduction

Hyponatremia with hyperkalemia is uncommon in infancy but represents a medical emergency due to the risk of life-threatening cardiac arrhythmia. While congenital adrenal hyperplasia (CAH) is a key differential diagnosis, other potential causes must also be considered, including hypoaldosteronism and pseudohypoaldosteronism (PHA) [1,2]. We report a case of secondary PHA in a six-month-old male patient who presented with recurrent vomiting, shock, severe hyponatremia, and hyperkalemia requiring intensive care. We also discuss the differential diagnoses of these biochemical abnormalities and summarize the key distinguishing features.

## Case presentation

A six-month-old male with an unremarkable birth history and normal newborn metabolic screening presented with weight loss, poor feeding, and recurrent vomiting. He had been last seen at the child health clinic two months ago for a routine check-up, which had been normal, and he had no prior history of hospitalization. His body weight on admission was 7.43 kg, reflecting a >10% decline from his previous weight of 8.45 kg two months earlier. There was no notable family history of similar illness or early infantile death, and the parents were not consanguineous. Physical examination showed significant dehydration with cool extremities and sunken eyes. Systemic examination was unremarkable, and there were no dysmorphic features, abnormal skin pigmentation, or genital abnormalities. Initial laboratory investigations revealed significant electrolyte disturbances, including severe hyponatremia of 119 mmol/L, hyperkalemia of 6.6 mmol/L, and metabolic acidosis with a bicarbonate level of 9.8 mmol/L. The serum chloride level was 90 mmol/L, and the calculated anion gap was 19 mmol/L. The serum lactate was normal. The initial glucose level was 7.6 mmol/L, with subsequent measurements remaining stable between 5.2 and 6.0 mmol/L. Kidney function was mildly impaired with elevated serum urea at 8.6 mmol/L and creatinine at 44 umol/L. His urinalysis showed pyuria, and urine culture subsequently grew Escherichia coli, confirming a diagnosis of UTI.

The patient deteriorated shortly after admission with worsening hypotension at 65/35 mmHg, tachycardia at 200 beats per minute, and respiratory distress requiring mechanical ventilation. Aggressive fluid resuscitation with isotonic crystalloid was initiated, and his electrolyte imbalances and acidosis were managed with intravenous sodium bicarbonate and dextrose-insulin therapy. Intravenous meropenem was administered for the UTI, with intravenous hydrocortisone started for the possible adrenal insufficiency pending further investigations. Investigations for CAH, including the serum cortisol, 17-hydroxyprogesterone, androstenedione, 21-deoxycortisol, and 11-deoxycortisol, returned within normal limits. However, the serum renin was elevated at 41.92 ng/ml/hour (reference range: 2.4-37.00 ng/ml/hour) with aldosterone markedly increased at 102,918 pmol/L (reference range: 56-1,943 pmol/L), suggestive of aldosterone resistance (Table 1).

**Table 1:** Initial investigations and subsequent work-up Initial investigations showed hyponatremia, hyperkalemia, and metabolic acidosis. Subsequent work-up revealed elevated renin and aldosterone levels

Variables	Results	Reference ranges
Initial investigations		
Sodium (mmol/L)	119	136–145
Potassium (mmol/L)	6.6	3.5–5.7
Chloride (mmol/L)	90	98–107
Urea (mmol/L)	8.6	1.4–6.8
Creatinine (µmol/L)	44	<37
pH	7.34	7.35–7.45
pCO_2_ (kPa)	2.5	4.7–6.0
HCO_3_ (mmol/L)	9.8	22–26
Base excess (mmol/L)	-13.3	-2.0–2.0
Further investigations		
Renin (ng/ml/hour)	41.92	2.40–37.00
Aldosterone (pmol/L)	102918	56–1,943
Cortisol (nmol/L)	6796	133–537
17-hydroxyprogesterone (nmol/L)	1.2	<8
Androstenedione (nmol/L)	<0.5	<2.4
21-deoxycortisol	<2.5	≤2.5
11-deoxycortisol	2.5	≤4.3

The patient's clinical condition improved markedly over the next five days, with the normalization of electrolytes and kidney function. His follow-up serum renin and aldosterone levels returned to normal a month later. Further imaging studies for his UTI revealed right-sided hydronephrosis with an anteroposterior renal pelvic diameter of 1 cm, and a hydroureter measuring 1.5 cm. A micturating cystourethrogram performed two months later showed no evidence of vesicoureteral reflux. His growth was normal in the following year, with no significant electrolyte disturbances noted during follow-up.

## Discussion

Secondary PHA is a rare condition, with an estimated annual incidence of 1 in 13,200 live births [3]. It predominantly affects infants and is most often associated with UTI and congenital anomalies of the kidney and urinary tract (CAKUT). While the exact pathophysiology of secondary PHA remains unclear, it is believed that the partial renal tubular insensitivity to aldosterone in infants may play a crucial role [4]. This effect may be further exacerbated by the local cytokine alterations during acute inflammation with UTI.

Previous studies have shown that secondary PHA developed in the setting of UTI in 90% of cases, of which 50-80% had CAKUT [5]. A study from Ireland by Kaninde et al. reported seven cases of transient PHA, all initially presenting with sepsis-like symptoms. All were later diagnosed with UTIs, and five had CAKUT, including two with high-grade bilateral vesicoureteral reflux (VUR) [3]. A review by Delforge et al. noted that most cases of secondary PHA occur in infants younger than six months, with VUR being the most commonly associated malformation [4]. Although the overall prognosis is generally good, secondary PHA can present acutely, and there have been reports of life-threatening complications such as cardiopulmonary arrest and ventricular fibrillation, underscoring the importance of early diagnosis and prompt management [1,2].

During the acute episodes, patients may present with signs and symptoms of hypoaldosteronism, typically poor feeding, hyponatremia, hyperkalemia, and metabolic acidosis. However, the symptoms can be very non-specific, highlighting the need for high vigilance when treating young infants with suspected UTI. Other important differential diagnoses to consider include CAH, side effects of medications (such as trimethoprim and renin-angiotensin-aldosterone inhibitors), and, less commonly, hypoaldosteronism and primary PHA. Table 2 outlines the characteristic features of these conditions.

**Table 2:** Features of secondary PHA and other differential diagnoses ACTH: adrenocorticotropic hormone; AD: autosomal dominant; AR: autosomal recessive; CAH: congenital adrenal hyperplasia; CAKUT: congenital anomalies of kidney and urinary tract; PHA: pseudohypoaldosteronism; UTI: urinary tract infection

	Secondary PHA	CAH	Primary PHA type 1	Hypoaldosteronism
Causes	Mineralocorticoid resistance, often exacerbated during inflammation or infection	Genetic; most commonly due to 21-hydroxylase deficiency	Genetic mutation affecting sodium channel (AR) or mineralocorticoid receptor (AD)	High renin: can occur due to withdrawal after prolonged mineralocorticoid therapy, critical illness, or isolated aldosterone synthase deficiency; low renin: associated with type 4 renal tubular acidosis, congenital causes
Clinical features	Failure to thrive, salt-wasting, often in association with UTI or CAKUT	Classical CAH: salt-wasting, hyperpigmentation, virilization, vomiting	Failure to thrive, salt-wasting; AR type can involve multiple organs, including recurrent lung infections or skin lesions	Variable presentation depending on causes or age
Investigations	Hyponatremia, hyperkalemia, metabolic acidosis			
	High renin and aldosterone; may have evidence of UTI and/or CAKUT	High renin, low aldosterone, high ACTH, low cortisol, high 17-hydroxyprogesterone (17-OHP), and androstenedione; hypoglycemia	High renin and aldosterone; AR: positive sweat and saliva tests with increased sodium and chloride concentrations	Low aldosterone with renin levels varying depending on the causes

Further investigations in our patient, including a normal level of 17-hydroxyprogesterone, made a diagnosis of CAH unlikely. Consistent with previously reported cases, our patient presented during the typical infantile period in the setting of UTI and the presence of CAKUT. The spontaneous resolution of elevated renin and aldosterone levels supports the diagnosis of secondary PHA. Although genetic testing could be considered to rule out primary PHA, we did not pursue it given the classical presentation and clinical course, and the absence of family history. Instead, we arranged a close follow-up to monitor the patient's growth and watch for any recurrence of symptoms.

The treatment of secondary PHA entails (i) fluid resuscitation using isotonic solutions with the correction of electrolyte imbalances and metabolic acidosis, (ii) antibiotics for UTI, and (iii) the evaluation and treatment for the possible underlying CAKUT. Serial electrolyte monitoring is crucial, and a rapid correction of hyponatremia should be avoided to prevent osmotic demyelination syndrome. An early use of corticosteroids may be reasonable when a differentiation between secondary PHA and CAH is challenging, especially in those presenting with shock. Fludrocortisone has been described as a potential therapy for secondary PHA [6], although its role has not been well established in large-scale studies. Aldosterone insensitivity may persist for some months after the resolution of infection or surgical correction of CAKUT [7], highlighting the importance of ongoing monitoring after hospital discharge. Clinicians should be mindful of this condition given its non-specific presentations and potentially life-threatening complications, particularly in young infants with suspected UTI or underlying CAKUT.

## Conclusions

Secondary PHA is a rare but significant cause of hyponatremia and hyperkalemia in infancy, often seen in patients with UTI and congenital anomalies of the urinary tract. Early recognition and prompt management are critical to prevent life-threatening complications such as shock and arrhythmia. Clinicians should maintain a high index of suspicion in infants presenting with hyponatremia and hyperkalemia, especially in the setting of UTI.
